# Impact of social isolation caused by the COVID-19 pandemic on the mood profile of active and sedentary older adults: physical activity as a protective factor

**DOI:** 10.3389/fpubh.2023.1221142

**Published:** 2023-10-02

**Authors:** Alexandro Andrade, Anderson D’Oliveira, Keyla Mara dos Santos, Ana Cecilia Rosatelli de Freitas Bastos, Stefano Corrado, Guilherme Torres Vilarino, Pierluigi Diotaiuti

**Affiliations:** ^1^Laboratory of Sport and Exercise Psychology, Department of Physical Education, Santa Catarina State University, Florianópolis, Brazil; ^2^Department of Human Sciences, Society and Health, University of Cassino and Lazio, Cassino, Italy

**Keywords:** physical exercise, mood states, mental health, public health, aged, SARS-CoV-2

## Abstract

**Background:**

The COVID-19 pandemic has changed our habits and lifestyle due to social isolation and mobility restrictions. This new scenario, together with the fear of contracting the coronavirus, influenced mental health, especially among older adults, who presented reductions in social contact and physical activity (PA). Thus, the objective of the study was to analyze the impact caused by social isolation during the COVID-19 pandemic on the mood states of active and sedentary older adults.

**Methods:**

This is an observational study conducted during the COVID-19 pandemic. Older adults aged over 60 years, who were registered in the database of the Secretariat for the Promotion of Citizenship from a city in southern Brazil, participated in the research. An online questionnaire was applied with questions about sociodemographic characteristics, level of PA, confinement, and mood states in two periods: May 2020 and June 2021. The Mann–Whitney U test was used to compare the mood states of active and inactive individuals during the pandemic.

**Results:**

One hundred and fifty participants answered the questionnaire, of which 80 (53.3%) reported practicing PA. More active older adults suffered fewer changes in mood when compared to inactive older adults, with lower levels of confusion (*p* = 0.035), depression (*p* = 0.002), and fatigue (*p* = 0.000). Older adults confined for more than 50 days were more likely to develop depression. In addition, the mood state correlated with the variable fear of contracting the coronavirus; the greater the fear, the greater the mental confusion, depression, fatigue, and tension, and the lower the vigor in the older adults. The practice of PA is also correlated with the mood state; the greater the number of hours dedicated to PA, the lower the confusion, depression, fatigue, and tension of the older adults.

**Conclusion:**

The practice of PA positively influenced the mental health of older adults during periods of isolation and social restrictions. PA has a protective factor for the development of mental health problems and improves mood states, with greater time performing PA leading to more benefits.

## Introduction

In March 2020, the World Health Organization (WHO) declared the COVID-19 pandemic a public health concern (1). Due to the high transmission rate of the disease, social isolation measures were used worldwide to reduce transmission of the virus and prevent associated diseases and deaths (2, 3).

The pandemic has presented new challenges for society. It has been shown that the COVID-19 pandemic is associated with higher levels of worry, fatigue, loneliness, avoidance, and covid-anxiety syndrome across different countries (4, 5). In Brazil, the first case of COVID-19 was in February 2020, but soon the cases began to grow, with Brazil being the second country with the most deaths from COVID-19 in the world (6). In this context, the older adult population was characterized as a risk group, with greater chances of developing more severe forms of the disease, requiring intensive care, due to a higher rate of hospitalizations and a higher incidence of deaths (7, 8). Thus, these factors directly implied the severity of the degree of isolation of this population (9), which negatively affected mental health and well-being (10), due to emotional stress factors, such as activity restrictions, mourning for family and friends, and conflicting information from social media (11).

As a result of these factors, significant increases in anxiety and depression rates were observed during this period (12). Deleterious effects were also observed in mood states, which are frequently temporary and can vary in intensity and duration (13). The ideal mood profile is known as the iceberg profile, characterized by a high level of vigor and low levels of tension, fatigue, anger, depression, and confusion (14, 15). Although researchers have reported that with advancing age, the mood profile tends to approach the iceberg profile, in the midst of confinement, negative changes were observed, which demonstrated that most mood variables suffered oscillations mediated by the restrictive measures of each moment (16).

The COVID-19 pandemic presented several challenges for the population due to lifestyle changes related to social isolation (17). Older adults, characterized as a risk group, presented significantly reduced practice of physical activity (PA) and social contact and were exposed to several psychological stress factors that negatively affected mental health (18, 19). Studies have revealed disturbances in the mood profile of the population, reporting increased levels of tension, depression, fatigue, anger, and confusion, as well as a reduction in vigor, compared to previously observed patterns (20).

In the context prior to the pandemic, physical inactivity was already considered a global health problem, being considered the fourth largest risk factor for mortality in the world and an economic burden for society (21, 22). In addition, the isolation related to COVID-19 negatively influenced health-related behaviors because, during this period, older adults presented a reduction in the quality of nutritional standards, increased alcohol consumption, and more expressively, significantly reduced practice of physical activity (11). This reduction was associated with an increase in sitting time, and reductions in the value of metabolic equivalents of task (METs), and the number of daily steps (23). While the practice of PA during the pandemic was associated with improved well-being, quality of life, and mood in general (24, 25), deprivation or reduced practice was associated with several negative effects on the physical and mental health of older adults, reinforcing the relevance of maintaining the practice of PA within the possibilities of low exposure to the virus in this context (26).

In this way, social isolation negatively influences mood states and the level of PA (27, 28), and lower levels of PA directly impact mood states (29), thus a vicious cycle is formed. However, the use of PA to interrupt this cycle has been shown to be efficient. In younger people (19–59 years), moderate PA during the isolation period improved mental health (30), being a strategy used in different populations even before periods of restriction. Considering the relevance of the topic and the lack of studies that relate mood in older adults and the practice of PA during social isolation due to the COVID-19 pandemic, the aim of the current study was to analyze the impact caused by social isolation during the COVID-19 pandemic on the mood states of active and sedentary older adults.

## Methods

### Study design and participants

This observational study was conducted using self-administered online questionnaires during two moments of the pandemic: May 2020 and June 2021, periods characterized by a higher and lower level of social isolation according to the restrictive measures of the Brazilian health authorities, respectively. The older adults were recruited from the databases of the Secretariat for the Promotion of Citizenship from a city in southern Brazil. A telephone contact was made to verify the interest in participating in the study. For those interested, a link was sent to access the questionnaire through Google Forms. People aged 60 or over of both sexes, residents of their own homes or family members, were included in the sample.

The study was approved by the Ethics Committee for Research Involving Human Beings of the State University of Santa Catarina, under number 40392220.2.0000.0118. The questionnaire was evaluated anonymously. The patients/participants provided their written informed consent to participate in this study. Sociodemographic data related to social isolation/confinement, level of PA, and mood states were collected.

### Sociodemographic and clinical aspects

Self-administered questionnaires were used to collect data on sociodemographic and clinical characteristics, including sex, age, marital status, and occupation, as well as PA, level of apprehension/fear about contracting COVID-19, and period of social isolation. The levels of PA were collected from the participants according to the question: How many hours a week do you dedicate to physical exercises and sports during the quarantine period? Participants were classified as inactive (no exercise) or active (performed physical exercise for at least 30 min a week). Confinement levels were collected with the following question: Approximately how many days have you been confined for? For the classification of confinement levels, the criterion based on the analysis of the likelihood test was used, thus the best classification used was up to 50 days confined and more than 50 days confined.

### Mood states

The Profile of Mood States (POMS) is one of the most used tools for assessing moods across various populations (31, 32). The Brunel Mood Scale (BRUMS), derived from the POMS, was used to assess mood states (tension, depressed mood, anger, vigor, fatigue, and confusion) (33). The BRUMS consists of 24 questions, with response options for each one ranging from 0 (none) to 4 (extremely), depending on the mood state at the time of assessment. The total score for each mood ranges from 0 to 16. The BRUMS has proven to be a valid and reliable tool to assess the mood state of Brazilian and the older adult population (33, 34).

## Statistical analysis

Data analysis was performed using the software Statistical Package for the social sciences (version 20.0), with descriptive statistics (mean, standard deviation, frequency, and percentage) and inferential statistics. The distribution of data normality was determined by the Kolmogorov–Smirnov test. The Mann–Whitney U test was used to compare the mood states of active and inactive individuals during the pandemic, in addition to comparing the mood of older adults at the beginning and end of social isolation. Spearman’s correlation test was used to verify the correlation between mood and the degree of social isolation. Data were stratified by age (over 70 years old or under 70 years old). The Mann–Whitney U test was used to compare age ratings. Factors associated with depression in older adults during the pandemic were analyzed using logistic regression. Thus, it was possible to estimate the crude and adjusted odds ratios (OR), as well as their respective 95% confidence intervals (95% CI). The independent variables were inserted according to the following hierarchical model: sex, marital status, and educational level in the first level, days of confinement and fear of contracting coronavirus in the second level, and physical activity in the third level. The hierarchical model is used when the choice of factors to be included in the model is based on a conceptual structure, which describes the hierarchical relationships between risk factors. This model is used to study the determinants of childhood infectious diseases, illnesses, malnutrition, low birth weight, infant mortality, hypertension and obesity (35). While the following examples are derived from the field of child health, the general principles apply to many other health issues as well. The hierarchical model was applied in this article with the objective of estimating the factors associated with the presence of mood state depression in the older adult during the pandemic. Socioeconomic factors are the distal determinants (gender, marital status, and education) and can affect, directly or indirectly, all other groups of risk factors. Second-level variables include, in turn, days of confinement and fear of contracting the coronavirus and can affect third-level variables, in this case physical activity (36).

For the categorization of variables, a likelihood test was used, as proposed by Bu et al. (37), using a proportion of 0.5. Variables were included in the adjusted model regardless of the *p*-value of the crude analysis. The significance level used in this study was *p* < 0.05.

## Results

A total of 255 older adults were invited to participate in the study, of which 150 answered the questionnaires, 83 in the first data collection and 67 in the second. Most of the older adults were women (88%) and were between 60 and 87 years old. Table 1 presents the sociodemographic characteristics of these individuals.

**Table 1:** Sociodemographic characteristics and physical activity of participants.

	Older adults (*n* = 150)
**Age (mean ± SD)**	68.60 ± 6.4
**Sex**	***N* (%)**
Female	132 (88%)
Male	18 (12%)
**Marital status**	***N* (%)**
Single	9 (6%)
Married	63 (42%)
Widower	52 (34.7%)
Separate	26 (17.3%)
**Do you currently practice your profession?**	***N* (%)**
Yes	28 (18.7%)
No	43 (28.7%)
Retired	79 (52.7%)
**Health insurance**	***N* (%)**
Yes	37 (45.1%)
No	45 (54.9%)
**How do you rate your health currently?**	***N* (%)**
Terrible	1 (1.2%)
Bad	2 (2.4%)
Regular	27 (32.9%)
Good	44 (53.7%)
Excellent	8 (9.8%)
**Days of confinement (Mean ± SD)**	101.52 ± 80.0
**Level of fear of contracting the coronavirus**	***N* (%)**
None	22 (14.7%)
Low	19 (12.7%)
Moderate	52 (34.7%)
Very high	40 (26.7%)
Extreme	17 (11.3%)
**Practice physical activity**	
Yes	80 (53.3%)
No	70 (46.6%)

SD = standard deviation; F = absolute frequency; % = relative frequency/percentage.

Older adults who remained active during the pandemic showed a more positive mood for mental health and had lower levels of confusion (*p* = 0.035), depression (*p* = 0.002), and fatigue (*p* = 0.000) compared to sedentary older adults (Table 2). When stratifying by age, there was a difference in depression (*p* = 0.034) and fatigue (*p* = 0.030) between active and sedentary older adults aged less than 70 years, in addition to a difference in depression between active and sedentary older adults aged over 70 years (*p* = 0.038).

**Table 2:** Mood states of active and sedentary older adults in the pandemic.

Mood states	Active older adults (*n* = 80) Mean ± SD	Sedentary older adults (*n* = 70) Mean ± SD	*p*-value
Tension	3.23 ± 2.5	4.21 ± 3.3	0.082
Depression	1.71 ± 2.2	3.37 ± 3.4	0.002*
Anger	1.36 ± 2.1	2.28 ± 3.2	0.179
Vigor	7.52 ± 3.5	6.91 ± 3.6	0.308
Fatigue	1.76 ± 2.1	3.94 ± 4.0	0.000*
Confusion	1.95 ± 2.2	1.95 ± 2.2	0.035*

SD = standard deviation; Mann–Whitney-U-test. *Statistical significance *p* < 0.05.

In addition, older adults who remained confined for more than 50 days were more likely (23 times) to develop mood depression (OR 0.23; CI 1.10–0.61) (Table 3).

**Table 3:** Factors associated with the presence of mood state depression in the older adults during the pandemic.

Variables	Brute analysis	Adjusted analysis**
	OR (CI95%)	
**Sex** ^a^		
Male	1.00 (0.33–2.97)	1.32 (0.39-4.41)
Female	1	1
**Marital status** ^a^		
Single	1.12 (0.22–5.66)	0.92 (00)
Married	0.79 (0.29–2.16)	1.10 (0.13–9.01)
Widower	0.67 (0.23–1.93)	0.79 (0.19–3.22)
Separate	1	1
**Educational level** ^a^		
Unlettered	1	1
Complete primary education	0.66 (0.07–5.67)	0.47 (0.11–1.98)
Incomplete primary education	0.31 (0.04–2.20)	1.08 (0.27–4.33)
Complete high school	0.66 (0.09–4.52)	1.35 (0.34–5.36)
Complete higher education	0.84 (0.12–5.49)	0.00 (0.00–0.00)
Postgraduate	0.00 (0.00–0.00)	1.54 (0.17–13.57)
**Days of confinement** ^b^		
Until 50 days	1	1
More than 50 days	0.25 (1.10–0.61)*	0.23 (0.09–0.59)*
**Fear of contracting coronavirus** ^b^		
None	0.85 (0.22–3.26)	2.06 (0.38–11.01)
Low	0.48 (0.11–2.15)	0.57 (0.08–3.65)
Moderate	0.53 (0.16–1.75)	0.99 (0.23–4.16)
Very high	0.67 (0.20–2.25)	0.95 (0.23–3.93)
Extreme	1	1
**Practice physical activity** ^c^		
Does not exercise	1.24 (0.60–2.55)	0.82 (0.31–2.14)
Practice exercise	1	1

**Analysis adjusted for all variables. *Hosmer-Lemeshow* = 0.758. *Significant association by confidence intervals (95% CI).

a Sex, marital status, and educational level.

b Days of confinement and fear of contracting coronavirus.

c Physical activity.

Older adults at the end of social isolation showed a reduction in confusion, depression, and fatigue when compared to the beginning of isolation (2020) (Table 4). In the stratification by age, there was a difference in fatigue (*p* = 0.022) in older adults under 70 years old, whereas in older adults over 70 years old, the differences were in the variables depression (*p* = 0.005) and fatigue (*p* = 0.048) between days of social isolation.

**Table 4:** Comparison of the mood state of the older adults in 2020 and 2021.

Mood	Social isolation 2020 (*n* = 82) Mean ± SD	Social isolation 2021 (*n* = 68) Mean ± SD	*p*-value
Tension	4.03 ± 2.9	3.25 ± 2.0	0.061
Depression	2.82 ± 2.9	2.11 ± 2.9	0.028*
Anger	2.03 ± 3.0	1.48 ± 2.4	0.161
Vigor	6.93 ± 3.4	7.64 ± 3.7	0.291
Fatigue	3.28 ± 3.4	2.17 ± 3.1	0.005*
Confusion	2.71 ± 2.6	2.02 ± 2.5	0.038*

SD, Standard derivation; *Statistical significance at *p* < 0.05.

Mann–Whitney U Test.

**Figure 1:**
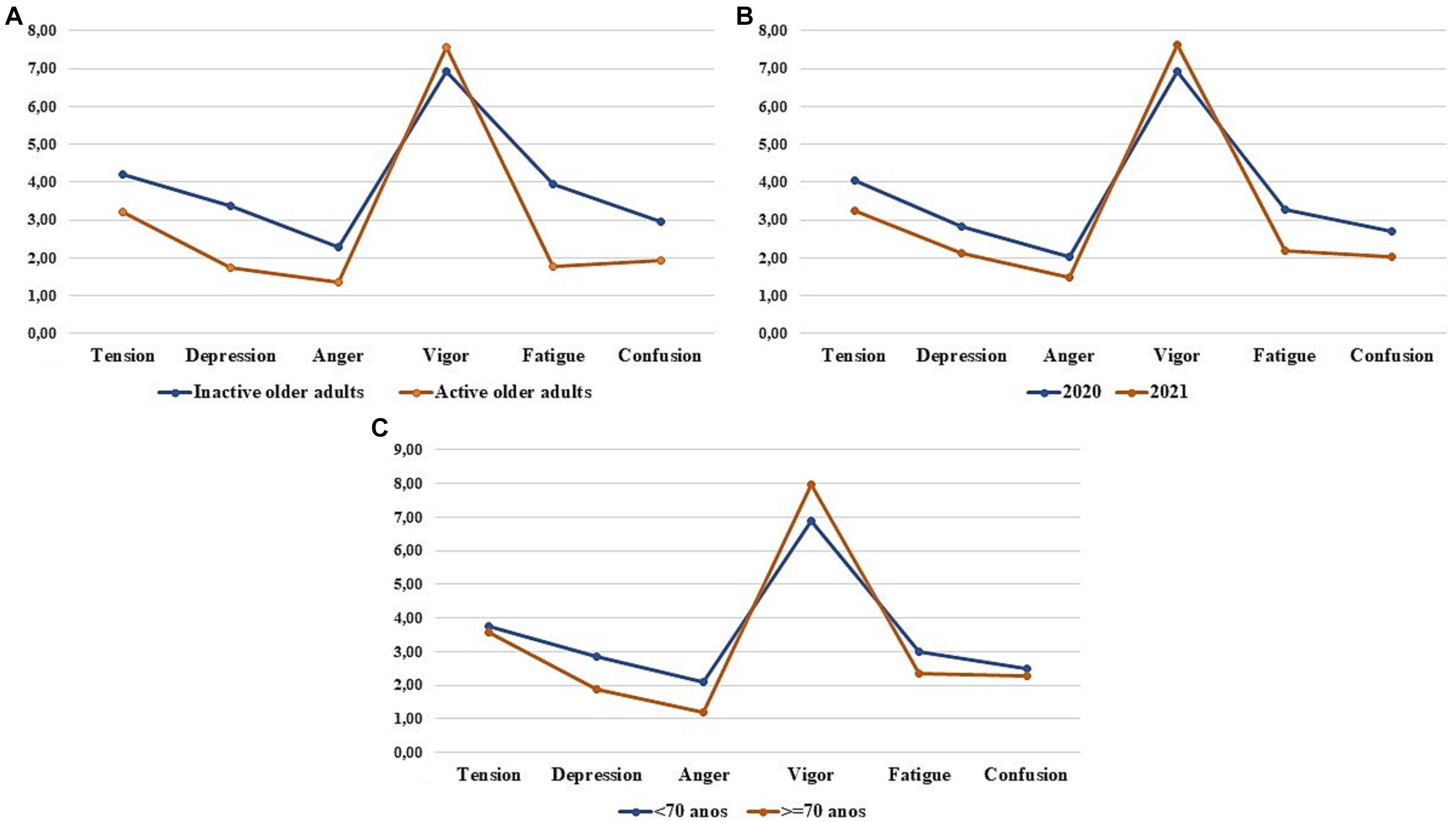
Mood profile of older adults according to physical activity (**A**), pandemic period (**B**) and age (**C**).

Figure 1 represents the iceberg profile identified in the older adults according to the practice of physical activity, the pandemic period, and age.

Correlations were observed between the degree of social isolation and mood in the older adults during the COVID-19 pandemic. The older adults who left home more frequently during the pandemic performed more hours of physical activity (*p* = 0.000; *r* = 0.487), and presented the following characteristics in terms of mood: less confusion (*p* = 0.022; *r* = −0.250), depression (*p* = 0.001; *r* = −0.358), fatigue (*p* = 0.019; *r* = −0.256), and tension (*p* = 0.014; *r* = −0.268) when compared to older adults with more days of social isolation.

Similar results were observed when stratifying by age. In older adults under 70 years of age, the variable number of times a week they left home correlated with depression (*p* = 0.031; *r* = −0.305) and vigor (*p* = 0.046; *r* = 0.283). In the older adults over 70 years of age, it was found that the greater the number of days they left home, the less the confusion (*p* = 0.003; *r* = −0.509), depression (*p* = 0.016; *r* = −0.421), and tension (*p* = 0.009; *r* = −0.457), suggesting that a greater the number of days in social isolation is associated with worsening mental health of the older adults.

The mood state also correlated with the variable fear that the older adults have of contracting the coronavirus; the greater the fear of contracting the coronavirus, the greater the confusion (*p* = 0.020; *r* = 0.189), depression (*p* = 0.026; *r* = 0.181), fatigue (*p* = 0.022; *r* = 0.186), and tension (*p* = 0.002; *r* = 0.251), and the lower the vigor (*p* = 0.033; *r* = −0.173) in older adults.

The practice of physical activity also correlated with mood states; the greater the number of hours dedicated to physical activity, the lower the mental confusion (*p* = 0.022; *r* = −0.244), depression (*p* = 0.000; *r* = −0.308), fatigue (*p* = 0.000; *r* = −0.372), and tension (*p* = 0.015; *r* = −0.197) of older adults (Table 5).

**Table 5:** Mood state correlations with days of social isolation, fear of contracting the coronavirus and practice of physical activity.

Mood states	Tension	Depression	Anger	Vigor	Fatigue	Confusion
Days of social isolation	*p* = 0.014*	*p* = 0.001*	*p* = 0.457	*p* = 0.488	*p* = 0.019*	*p* = 0.022*
	*r* = −0.268	*r* = −0.358	*r* = 0.067	*r* = −0.063	*r* = −0.256	*r* = −0.250
Fear of contracting the coronavirus	*p* = 0.002*	*p* = 0.026*	*p* = 0.476	*p* = 0.033*	*p* = 0.022*	*p* = 0.020*
	*r* = 0.251	*r* = 0.181	*r* = 0.058	*r* = −0.173	*r* = 0.186	*r* = 0.189
Practice of physical activity	*p* = 0.015*	*p* = 0.000*	*p* = 0.180	*p* = 0.309	*p* = 0.000*	*p* = 0.022*
	*r* = −0.197	*r* = −0.308	*r* = −0.109	*r* = 0.083	*r* = −0.372	*r* = −0.244

*Statistical significance at *p* < 0.05. Spearman correlation.

## Discussion

During the COVID-19 pandemic, an increase in the prevalence of mood disorders was observed due to social isolation and fear of contracting the disease (10, 20, 38), allied with a reduction in the level of PA (26). However, the practice of PA has been shown to mitigate the deleterious effects of confinement on mental health (25).

In the present study, less confined older adults who left the house on more days performed more PA and presented a better mood profile, with lower levels of mental confusion, depression, fatigue, and tension, when compared to the more confined older adults, reinforcing the associations between confinement, PA, and mental health, already evidenced in other studies (39, 40).

### Relationship between confinement and mental health

According to Santomauro et al. (41) the reduction in human mobility was considered one of the biggest factors associated with the emergence of mood disorders during the pandemic. In the study conducted by Richardson et al. (16) with older adults in the United Kingdom, depressive mood increased until the beginning of the easing of restrictive measures, reinforcing the possible relationship between isolation and mental health. In our study, older adults who were confined for more than 50 days were 23 times more likely to develop a depressed mood. When analyzing the mood profile during different periods of the pandemic, it was observed that at the end of 2021, the older adults presented a better mood profile (less confusion, depression, and fatigue) than in 2020.

Factors such as fear, uncertainty, economic hardship, and changes in daily habits showed a negative correlation with the general population’s mental health (42). Fear of COVID-19 has been shown to be significantly associated with worsening mental health status, negatively influencing factors such as anxiety, stress, depression, and sleep quality (43). In our study, the variable fear of contracting the coronavirus was positively correlated with confusion, depression, fatigue, and tension, and negatively correlated with vigor in the older adults, thus demonstrating that individual concern related to COVID-19 potentiated the deleterious effects of confinement in older adults. This result may be associated with the fact that due to the fear of contracting COVID-19, many older adults were isolated, which reduced the level of PA.

### Mood profile and levels of physical activity

The practice of PA during the pandemic seems to be a factor that promotes mental health and protects against depression, fatigue, and confusion in mood in older adults.

PA was already recommended for the older adult population before the outbreak of COVID-19, as it helps to maintain physical and mental health, in addition to being considered a form of treatment for various diseases and health problems (44–49). Furthermore, PA can help improve the quality of life (50, 51) and psychological well-being associated with positive mood indices (52, 53). Therefore, during the pandemic, maintaining PA levels was essential to reduce the damage caused by isolation. However, a reduction in PA was observed in the general population, including among older adults (26).

PA proved to be fundamental during the pandemic, as active older adults had lower levels of confusion, depression, and fatigue. Analyzing other factors, the practice of PA continued to show positive results. When comparing older adults over and under 70 years of age, we found that the younger group presented more benefits with the practice of PA, since among those over 70 years old there were significant improvements only in depression. With increasing age, it is expected that older adults will have more difficulties in moving and performing exercises, which may explain the differences between the groups since the PA practice was not controlled. In the study carried out by Sojli et al. (54), emotional stress related to COVID-19 was similar in individuals aged 65–75 years or older and relatively low when compared to other age groups, however, the practice of PA was not analyzed.

Corroborating the findings related to the protective effect of PA on the mental health of older adults, other studies observed a relationship between PA and depressive symptoms (55, 56). In addition, a review showed that higher levels of PA in volume, frequency, and regularity are associated with lower levels of symptoms of depression and anxiety in the general population (57). Important dose-response relationships that enhance the protective effect were also observed through the negative correlation between the number of hours dedicated to PA and the mood profile observed in the older adults in the variables confusion, depression, fatigue, and tension.

The WHO recommends the practice of at least 150 min of PA per week and reinforces that any level of PA greater than none can be beneficial to health when compared to physical inactivity (58). In the current study, it was observed that 30 min of PA or more were enough to improve the mental health of the older adults during the pandemic, reinforcing the relevance of protection and promotion that increased levels of PA provided. These results are in line with scientific evidence, since PA presents, through various functions such as psychological, physiological and immunological, a vital component for the health of different populations, one of them investigated in the present study as the older adults (59, 60). In this regard, home-based physical exercise programs for older adults seem to offer a safe, easily accessible, and low-cost PA alternative for this population (61).

### Limitations and future studies

Despite the important results verified, this study has some limitations, such as the small sample size, the sample being from a single municipality, and the majority of participants being female. In addition, it was not verified whether the participants had mental health problems before the pandemic and the intensity of the practice of PA was not evaluated.

Future studies should be developed, including monitoring variables regarding the type of physical exercises, volume, intensity, duration, and weekly sessions, among others, in addition to expanding mental health variables during the post-pandemic process of COVID-19.

Future studies should try to measure the level of daily activity of the participants, considering that activities of daily living (for example, gardening, sweeping the house, among others), can also be considered physical activities, depending on the amount performed weekly, and may also impact the physical and mental health of participants.

Another point to be considered as a limitation is the level of distraction that the participants had, when they did not remain in isolation, since in our findings, the older adults who left home more presented a better mood profile, compared to the group with more days in social isolation. In new studies, it would be interesting to identify the level of distraction or interaction when the older adults do not remain in social isolation (outside their homes), in order to identify if there are any additional relationships, considering the greater chance of social contact and even levels of daily activities, affecting physical activity. In addition, future studies should consider the role of possible pharmacological or psychological treatments.

Studies with proposals and applications of different physical exercise protocols for older adults are recommended, both at the individual level and for future public policies.

### Strengths, innovations, and applications of the study

Our study is one of the rare empirical investigations on this subject, being innovative as it presents a comprehensive view of changes in the mood of older adults during the pandemic associated with the practice of PA. One of the strengths of the study is the analysis of the time of social confinement to which many older adults were subjected during the pandemic, its effects on mood and mental health, and the role of PA in this context. Relevant aspects of the effects of social confinement and the possible protective effect of PA on the mental health of older adults in a pandemic context were demonstrated.

As applications of the findings, we can highlight the need for attention to the time of social isolation that older adults are subjected to, during pandemics or even in social isolation contexts, to reduce these periods as much as possible and provide options for PA remotely, face-to-face, or with the use of technologies, seeking to motivate and promote PA for older adults, knowing the benefits to physical and mental health. These findings can be used as a source of inspiration to guide public policies.

## Conclusion

The practice of PA positively influences the mental health of older adults during periods of isolation and social restrictions. PA, for older adults in social isolation, seems to be a protective factor against the development of mental health problems, and may improve mood states, especially depression, fatigue, and confusion. Exposure to long periods of social confinement is a high-risk factor for mood and mental health problems, especially when associated with a sedentary lifestyle.

## Data availability statement

The raw data supporting the conclusions of this article will be made available by the authors, without undue reservation.

## Ethics statement

The studies involving humans were approved by Ethics Committee for Research Involving Human Beings of the State University of Santa Catarina. The studies were conducted in accordance with the local legislation and institutional requirements. The participants provided their written informed consent to participate in this study.

## Author contributions

AA contributed to the design and coordination in the production of the study. AA, AD’O, and GV contributed to data acquisition, data analysis and interpretation. AA, AD’O, GV, SC, and PD critically reviewed the article for intellectual content. KS contributed to data analysis and interpretation. AB, AD’O, and GV drafted the manuscript. All authors contributed to the article and approved the submitted version.
